# Dissection of Highly Prevalent *qnrS1*-Carrying IncX Plasmid Types in Commensal *Escherichia coli* from German Food and Livestock

**DOI:** 10.3390/antibiotics10101236

**Published:** 2021-10-12

**Authors:** Katharina Juraschek, Annemarie Käsbohrer, Burkhard Malorny, Stefan Schwarz, Diana Meemken, Jens André Hammerl

**Affiliations:** 1 Department Biological Safety, German Federal Institute for Risk Assessment (BfR), Max-Dohrn Str. 8-10, 10589 Berlin, Germany; annemarie.kaesbohrer@bfr.bund.de (A.K.); Burkhard.Malorny@bfr.bund.de (B.M.); 2 Unit for Veterinary Public Health and Epidemiology, University of Veterinary Medicine, Veterinaerplatz 1, 1210 Vienna, Austria; 3 Department of Veterinary Medicine, Institute of Microbiology and Epizootics, Freie Universität Berlin, 14163 Berlin, Germany; stefan.schwarz@fu-berlin.de; 4 Department of Veterinary Medicine, Institute of Food Safety and Food Hygiene, Freie Universität Berlin, 14163 Berlin, Germany; Diana.Meemken@fu-berlin.de

**Keywords:** *E. coli*, *qnrS1*, IncX, fluoroquinolones, plasmids, transferability

## Abstract

Plasmids are mobile genetic elements, contributing to the spread of resistance determinants by horizontal gene transfer. Plasmid-mediated quinolone resistances (PMQRs) are important determinants able to decrease the antimicrobial susceptibility of bacteria against fluoroquinolones and quinolones. The PMQR gene *qnrS1*, especially, is broadly present in the livestock and food sector. Thus, it is of interest to understand the characteristics of plasmids able to carry and disseminate this determinant and therewith contribute to the resistance development against this class of high-priority, critically important antimicrobials. Therefore, we investigated all commensal *Escherichia* (*E*.) *coli* isolates, with reduced susceptibility to quinolones, recovered during the annual zoonosis monitoring 2017 in the pork and beef production chain in Germany (n = 2799). Through short-read whole-genome sequencing and bioinformatics analysis, the composition of the plasmids and factors involved in their occurrence were determined. We analysed the presence and structures of predominant plasmids carrying the PMQR *qnrS1*. This gene was most frequently located on IncX plasmids. Although the *E. coli* harbouring these IncX plasmids were highly diverse in their sequence types as well as their phenotypic resistance profiles, the IncX plasmids-carrying the *qnrS1* gene were rather conserved. Thus, we only detected three distinct IncX plasmids carrying *qnrS1* in the investigated isolates. The IncX plasmids were assigned either to IncX1 or to IncX3. All *qnrS1*-carrying IncX plasmids further harboured a β-lactamase gene (*bla*). In addition, all investigated IncX plasmids were transmissible. Overall, we found highly heterogenic *E. coli* harbouring conserved IncX plasmids as vehicles for the most prevalent *qnr* gene *qnrS1*. These IncX plasmids may play an important role in the dissemination of those two resistance determinants and their presence, transfer and co-selection properties require a deeper understanding for a thorough risk assessment.

## 1. Introduction

The World Health Organization (WHO) recognizes fluoroquinolones (FQ) as critically important antimicrobials (CIA) for the treatment of human infections [1,2]. Further resistance development against antimicrobials of this class should thus be avoided. The food production chain, starting from livestock and ending at the food product, plays an important role in the transmission of antimicrobial-resistant microorganisms, as well as for their evolution and dissemination [1,3,4]. *Escherichia* (*E*.) *coli*, a commensal species of the gastrointestinal tract of animals and humans, is a suitable indicator organism for monitoring the emergence of genes, leading to antimicrobial resistance (AMR) in gram-negative bacteria [5,6]. *E*. *coli* is also a common reservoir for mobile genetic elements (MGEs), such as plasmids, involved in the dissemination of genetic information to other commensal or pathogenic enteric microorganisms [7]. The screening of antimicrobial-resistant *E. coli* from livestock and food is widely established to estimate the prevailing AMR situations and dynamics over time.

Plasmids and other MGEs are major contributors to the spread of genetic information by horizontal gene transfer [8,9,10]. In general, they support the evolution and diversification of bacteria for e.g., developing resistances or novel pathotypes. In bacteria, horizontal gene transfer is mainly attributed to the spread of MGEs as gene cassettes, transposons, integrative conjugative elements (ICEs), and plasmids [11,12,13,14]. For the spread of FQ resistances, plasmid-mediated quinolone resistance (PMQR) genes play a major role. PMQR genes are notified to be substantially involved in the spread of FQ resistance in livestock [7,8]. Furthermore, several PMQR genes (*qnr*, *aac*(6′)-*lb-cr*, *qepA* and *oqxAB*) are known to be associated with a decrease in susceptibility against FQ. *qnrS1* especially was frequently reported as transmissible FQ-resistance gene in *E. coli* from food and livestock [15,16,17,18,19,20,21]. *qnrS*1 is of special concern, as this gene is often reported to be co-localized with resistance genes against extended spectrum cephalosporins (ESC) or resistance determinants to other antimicrobial classes. This gene has been shown to occur on plasmids carrying *bla*_CTX-M_ genes [6,8,16,18,22]. The co-occurrence of antimicrobial resistance genes on plasmids can lead to a long-term persistence of these elements by antimicrobial co-selection, which provides not only a selective advantage but also promotes their spread [10,23]. Qnr proteins are known to be associated with low-level resistances against FQ [24]. However, the genes are recognized for facilitating the selection of high-level FQ resistance in gram-negative bacteria [1,25,26]. Moreover, it has been shown that isolates carrying PMQR genes support the alteration of chromosomal sequences also involved in FQ resistance development [1,27,28,29,30]. Thus, further information on the occurrence of *qnrS1* in livestock and food will support a deeper understanding of potential sources of this determinant, mechanisms involved in its dissemination and the diversity of associated plasmids. Plasmids are mainly subdivided on the basis of specific incompatibility sequences (*inc* groups). This classification takes into account their stable co-residence in the same bacterial cell without any selection pressure [9,31]. The determination of predominant plasmid types will provide further information on the impact of specific MGEs in the spread of *qnrS1* and will help to evaluate the risk of FQ resistance development in other compartments, such as the human sector.

This study aims to identify prevalent *inc* plasmid types carrying *qnrS1* originating from the German livestock and food sector for the beef and pork production chain. We aimed to determine the genetic basis of elements involved in FQ resistance development, and to derive the core plasmid backbones of predominant *qnrS1*-carrying plasmids by whole-genome sequencing (WGS) and bioinformatics analysis. Furthermore, the commonalities and dissimilarities of the most prevalent *qnrS1*-carrying plasmids were depicted. Such studies will help to evaluate potential evolutionary processes associated with the occurrence and spread of *qnrS1*-carrying plasmids. Further, the conjugation ability of those plasmids was investigated to better assess the likelihood of *qnrS1* transmission. 

## 2. Results and Discussion

### 2.1. qnrS1 Is Highly Prevalent on IncX Plasmids in Commensal E. coli

Out of the investigated *E. coli* from livestock and food, *qnrS1* was the most prevalent PMQR gene. Of 2799 *E. coli* isolates obtained during the German monitoring programs in 2017, we identified 391 isolates representing a non-wildtype against ciprofloxacin (MIC ≥ 0.06 µg/mL) and/or nalidixic acid (MIC ≥ 16 µg/mL). PCR amplification revealed that 97 isolates carried *qnrS1*, while all other *qnr* determinants were detected only sporadically. S1-PFGE of *qnrS1*-positive *E. coli* coupled with Southern-blotting and DNA-DNA hybridization indicated that 85 isolates carried the *qnrS1* gene on a plasmid. The 12 chromosomally encoded *qnrS1* genes were detected in isolates representing eight distinct multi-locus sequence types (STs). This observation suggested a high heterogeneity of the *E. coli* carrying this PMQR gene within the chromosome. We found *qnrS1* to be the most frequent PMQR gene in isolates from the here investigated veal and pork source, as investigated in the monitoring program in 2017 (Table 1). To assign the plasmids to specific *inc* groups, we mapped the WGS data to all available *qnr* plasmid genomes published on NCBI as references, using the plasmidID tool. By this analysis, a high heterogeneity of *qnr* plasmids was detected. Out of our investigated WGS data of our isolates, two main clusters represented by IncY (n = 19) and IncX (n = 29) plasmids were determined. However, the *E. coli* comprising *qnrS1*-carrying IncX-plasmids were found to be highly diverse. The corresponding isolates exhibiting diverse STs were from different origins and exhibited distinct resistance profiles. Based on the XbaI-macrorestriction profiles, the high diversity of *E. coli* could be confirmed (data not shown), indicating that the occurrence of *qnrS1*-positive isolates is mainly triggered by the transmission of *qnrS1*-carrying plasmids. However, 23 of the 29 investigated *E. coli* with a *qnrS1* on an IncX plasmid were phenotypically resistant against ampicillin, demonstrating the potential link of *qnrS1* and *bla* genes.

The data of our analysis are in good agreement with previously published results. Similar to our observation of calf and pig isolates, *qnrS1* was also identified as the most prevalent PMQR gene in *E. coli* from investigated turkeys, broilers and layers worldwide [15,20,21]. Based on the combination of our results and the prevailing literature, *qnrS1* seems to be the most frequent PMQR gene in farm animals. It also seems that there is a strong association of *qnrS1* to IncX plasmids. Several plasmids of this incompatibility group have been described as efficient carriers of this gene in *E. coli* [1,8,16,19,32,33,34]. Dolejska et al. [6] further detected *qnrS*-carrying IncX plasmids in other sources i.e., horses, environmental samples and flies at an equine clinic.

In this study, *qnrS1* was found to be the most prevalent PMQR gene in livestock and food, frequently associated with plasmids of the IncX group. The *qnrS1*-carrying IncX plasmids were found to be disseminated among different *E. coli* STs recovered from various sources. As such, plasmids are often found in various genera or species of the Enterobacteriaceae, the main routes of transmission and spread need to be determined. To assess this further, in silico analysis of the genomes was performed to achieve deeper knowledge on the evolution of the plasmids, their stability and its dissemination.

### 2.2. Three Prevalent IncX Plasmids, Carrying qnrS1 in German Livestock Were Detected

The investigation of the *qnrS*1 IncX genomes resulted in the detection of three distinct reference plasmids representing the most frequent plasmid types present in German livestock in 2017. Table 2 includes the phylogenetic relationship of the plasmids. Therewith, the short-read sequences of only one isolate resemble the unnamed reference plasmid of strain R1701 (NZ_CP039972.1, *Klebsiella pneumoniae*). All other reference plasmids are represented by 14 (NZ_CP020088.1, unnamed plasmid identified in *Shigella flexneri*), eight (NZ_CP037995, psg_ww281 plasmid identified in *Salmonella enterica* subsp. *enterica* serovar Brancaster) and six (NZ_CP031373.1, pKpvST101_6 plasmid identified in *Klebsiella pneumoniae*) WGS datasets from our study. In general, the plasmid sequences are highly conserved (94% to 100% mapped) in comparison to their reference plasmids, indicating that only a minor evolutionary adaption prevails. All of the most frequently detected reference plasmids were larger in size and carried a *bla* gene, as presented in Table 2.

Dolejska et al. [35] emphasized the correlation between these IncX plasmids comprising *bla*_TEM_ and *bla*_CTX-M-15_ genes in association with *qnrS*, resulting in ESBL-producing *E. coli*. The frequent observation of *qnrS1*-carrying plasmids comprising ESBL-enhancing resistance genes stresses the necessity of thorough screening and a better characterization of *qnr*-positive *E. coli* for risk assessment. Furthermore, Guo et al. [36] described an IncX plasmid carrying a mobile colistin resistance gene (*mcr*). Thus, IncX plasmids seemed to be a potential reservoir for diverse combinations of resistances, decreasing the susceptibility against clinically important antimicrobials and antimicrobials of the last resort. IncX plasmids have regularly been described as a group harbouring *qnrS*1. Therefore, we decided to dissect this group of plasmids even further. 

#### 2.2.1. The Genomes of Prevalent *qnrS1*-Carrying IncX Plasmids 

In general, IncX plasmids can be assigned to six distinct subgroups, namely IncX1 to IncX6 [37,38]. Here, we only detected IncX1, IncX3 or a combination of both as carriers for *qnrS1*. Overall, the unnamed reference plasmid of the strain 0670 (NZ_CP020088) was the most prevalent IncX plasmid type (WGS data of 14 isolates) detected to carry a *qnrS1* gene.

#### 2.2.2. Characteristics of Plasmids Assigned to the Unnamed Reference Plasmid of the Strain 0670 

**Figure 1:**
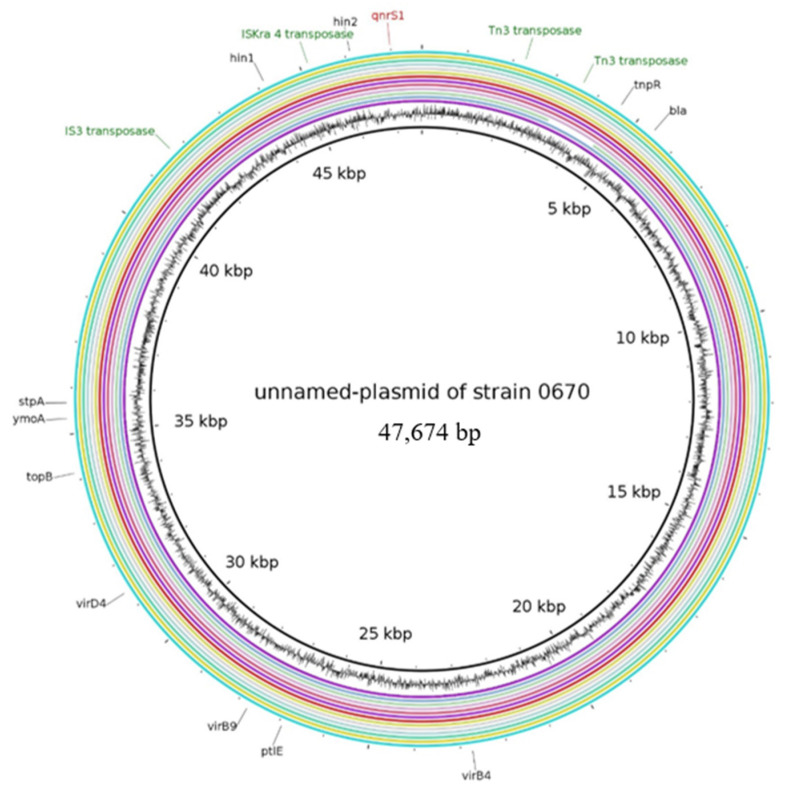
BRIG image of sequence contigs of individual isolates assigned to the unnamed reference plasmid (NZ_CP0200800). Contigs belonging to the isolates as indicated (from inner to outer ring): unnamed-plasmid of strain 0670, 17-AB00542, 17-AB00639, 17-AB00742, 17-AB00995, 17-AB01105, 17-AB01352, 17-AB01539, 17-AB01752, 17-AB01792, 17-AB01795, 17-AB01969, 17-AB02090, 17-AB02707 and 17-AB02951.

Twelve out of 14 WGS datasets resemble the reference plasmid under the number NZ_CP020088 (Figure 1). Due to the frequent occurrence of these plasmids, we can conclude that its genome structure represents the most prevalent *qnrS1* plasmid of *E. coli* from German livestock in 2017. The plasmid is 47,674 bp in size and harbours an IncX1 (100%) and an IncX3 (80.59% identical to NZ_CP020088) replicon sequence. Further, the resistance determinants *qnrS1* and *bla*_TEM-1_ are present on the reference plasmids, as well as on our detected plasmids.

The unnamed plasmid (NZ_CP020088) originates from a *Shigella flexneri* isolate recovered in Hangzhou, China from human origin. Comparable plasmids were shown to be spread worldwide, as close relatives were detected i.e., in *E. coli* from turkey meat (LR882060) or chicken meat (MK965545) in Norway and Brazil, respectively. Resistance determinants and associated IS elements or transposases of these plasmids are located in a single DNA region of approx. 15 kb. Downstream of *qnrS1*, the *hin* DNA-invertase was detected, which was in vicinity to a IS*Kra4* and a Tn*3* transposase. Upstream of the Tn*3* transposase, the *bla*_TEM-1_ gene is located. Outside of the resistance-IS region, different components of the type IV secretion systems (*virB4*, *virD4*, *ptlE*, *virB9*) were detected. The plasmid of 17-AB00639 lacks a 1325 bp DNA region, which encoded an additional Tn*3* family transposase present on the reference plasmid. Transmissibility evaluation using the mob-suite for these plasmids yielded an assignment of self-transmissibility (conjugative). All plasmids carried the MOB_P_ relaxase and the MPF_T_ mating pair formation (*mpf*) region. In vitro filter mating experiments demonstrated that all *qnrS*1 IncX-like plasmids were self-transmissible among *E. coli* at 37 °C. Verification of the plasmid structure within the *E. coli* J53 recipient showed no obvious differences between the plasmids of the donor strains and the transconjugants by PFGE and DNA-DNA hybridization. We thus conclude that the plasmids seemed to be genetically stable.

A frequent occurrence of *qnrS1* and *bla*_TEM-1_-carrying InX1 plasmids was previously described by Dobiasova and colleagues [39]. They found the presence of these plasmids in Enterobacteriaceae from food-producing animals and wildlife in Europe. Therewith, the combined existence of *qnrS1* and *bla*_TEM-1_ was mentioned as common. Furthermore, the highly conserved backbone consisting of *taxC* (relaxase encoding gene), *qnrS1* and *bla*_TEM_ of these plasmids was discussed. Due to the detection of 12 closed plasmid structures out of 14 matching plasmids, this study confirms the frequent occurrence of this conserved plasmid structure. The co-occurrence of IncX1 and IncX3 replicons represents a multi-replicon type that might be beneficial for the plasmid as it is useful for stable replication in isolates carrying either IncX1 or IncX3 plasmids. This plasmid structure again represents a possible evolvement for the dynamics of the *qnrS1* plasmid dissemination. All 14 plasmids carried a *pir* gene (encoding for replication initiation) and the type IV secretion system, necessary for conjugational transfer. A similar high conservation of the plasmid backbone was also described before [40]. The DNA-invertase gene *hin* as well as both resistance determinants were present in all detected plasmids matching to the reference plasmid. However, two plasmids comprised slightly altered structures indicating a possible hot spot for further evolutionary adaptions or acquisition of further resistance determinants. 

#### 2.2.3. Characteristics of Plasmids Assigned to the Reference Plasmid pKpvST101_6 

**Figure 2:**
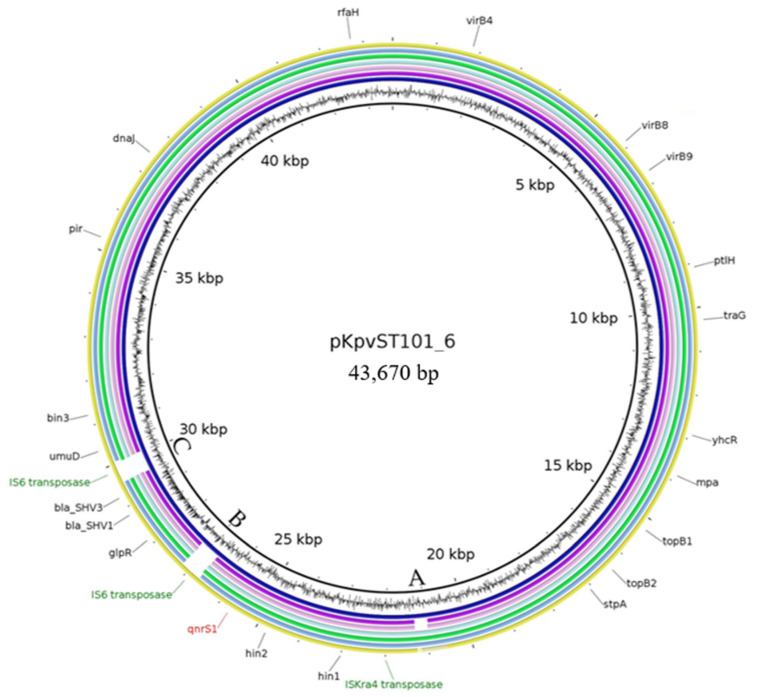
BRIG image of sequence contigs of individual isolates assigned to the reference plasmid pKpvST101_6 (NZ_CP031373). Contigs belonging to the isolates as indicated (from inner to outer ring): pKpvST101_6, 17-AB01005, 17-AB01006, 17-AB01018, 17-AB01798, 17-AB02071 and 17-AB02673. Regions lacking within the investigated are indicated by the capital letters A, B and C.

Plasmid pKpvST101_6-like (NZ_CP031373) structures were detected in six WGS datasets of the investigated isolates. pKpvST101_6 is 43,670 bp in size and carries an IncX3 replicon. The DNA region encoding the IS elements (IS*6*, IS*Kra4*), transposases and resistance determinants is 8.5 kb in size (Figure 2). This region includes an IS*Kra4* transposase gene, followed by the DNA-invertase genes *hin1* and *hin2*, and *qnrS1* encoding the pentapeptide repeat protein. Right after this structure the *bla*_SHV_ genes are present, flanked by the IS*6* transposase gene on each site. Outside of the resistance determinant carrying DNA region, the plasmid harbours the type IV secretion system genes *ptlH*, *virB4* and the conjugational transfer gene *traG*.

This plasmid was first detected in a *Klebsiella pneumoniae* strain from a hospital in the United Kingdom (CP031373.2). Similar plasmids were reported from an *E. coli* of poultry origin from the Netherlands (KX618696.1) and from *Citrobacter freundii* of a healthcare environment in Spain (MT720906.1). The plasmids detected in our study lacked certain regions compared to the reference plasmid, except for the plasmid occurring in isolate 17-AB02673. All other datasets were lacking two regions (B and C in Figure 2). Furthermore, the pKpvST101_6-like plasmids of 17-AB01005 and 17-AB01006 lacked an additional region. This included two IS*6* transposase genes, which are located downstream and upstream of the *bla*_SHV_. The area A is missing in the two plasmids detected in 17-AB01005 and 17-AB01006 (Figure 2). However, it did not cover a CDS. This missing sequence was located next to the IS*Kra4* transposase gene. Besides the reference plasmid pKpvST101_6, also the reconstructed plasmids out of the livestock isolates were assigned to be self-transmissible using the mob-suite. Furthermore, this prediction could be experimentally confirmed by in vitro filter mating studies leading to an efficient self-transfer between donor and recipient *E. coli*.

IncX3 plasmids, as carriers for *qnrS1* and *bla*_SHV_ genes, have been described as common in central Europe [39] and China [41]. Especially the presence of IS*26* (IS*6* family transposase) in the vicinity to the *bla*_SHV_ gene was notified before [42] and described as mobilizing-factor for the β-lactam resistance gene. Moreover, IncX3 plasmids have been described as carriers of carbapenem resistance genes, such as *bla*_NDM_, in clinical environments [43,44]. Thus, such plasmids play an important role in the dissemination of resistances against last resort antimicrobials. Furthermore, several IncX3 plasmids have been reported to carry *qnrB*, *qnrS* and *bla* genes [41,42,44,45], highlighting the importance of this plasmid for the dissemination of antimicrobial resistance genes in Enterobacteriaceae [37]. In contrast to the published results, the majority of our IncX3 plasmids of this study lacked the IS*6* transposase gene. In addition, a non-coding area next to the IS*Kra4* transposase gene was not detected in two plasmids, while present in the reference plasmid. This could suggest the alteration of the plasmid in its resistance determinant area. Therefore, as conjugative plasmid, carrying two important resistance determinants, the complete structure of this IncX3 plasmid should be investigated further.

#### 2.2.4. Characteristics of Plasmids Assigned to the Reference Plasmid psg_ww281 

**Figure 3:**
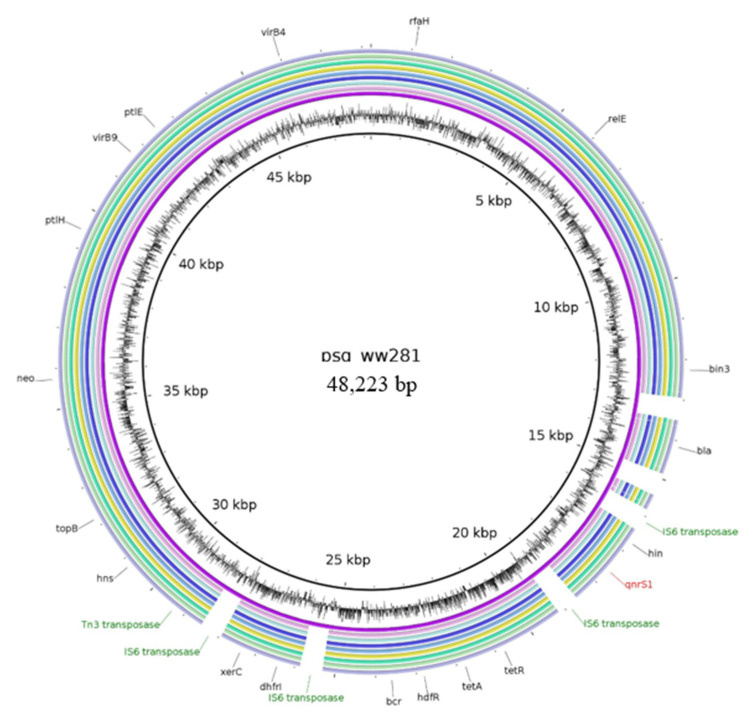
BRIG image of sequence contigs of the individual isolates assigned to the reference plasmid psg_ww281 (NZ_CP031373). Contigs belonging to the isolates as follows (from inner to outer ring): psg_ww281, 17-AB00544, 17-AB01619, 17-AB01686, 17-AB01707, 17-AB01875, 17-AB02711, 17-AB02721 and 17-AB02726.

Another frequently detected plasmid type matched to the reference plasmid psg_ww281 (NZ_CP037995). It was recognized as an IncX1 plasmid of 48,223 bp. This plasmid carried multiple resistance genes, including *aph(*3*′)-Ia*, *bla*_TEM-176_, *dfrA14*, *floR*, *qnrS1*, and *tet*(A). Thus, it confers phenotypic resistance against antimicrobials of different classes. In total, eight WGS datasets of the livestock isolates resemble this reference plasmid. As shown in Figure 3, the best-matching plasmids lacked certain regions in comparison to the reference plasmid psg_ww281.

For the first time, this reference plasmid was reported in a *Salmonella enterica* from a wet market in Singapore. A close relative of psg_ww281 was also found in Singapore, but occurred in an *E. coli* (plasmid pSGMCR103 (MK731977.1)). Later on, a similar plasmid was described from an *E. coli* of the Czech Republic (plasmid pCE1594 (MT859327.1)). On the reference plasmids, the resistance determinants are scattered within a DNA region ranging between 12 to 30 kb (Figure 3). The core genome of this plasmid type carries the resistance gene *bla* followed by an IS*6* transposase gene, downstream followed the DNA-invertase gene *hin*, the PMQR gene *qnrS*1 followed by another IS*6* transposase gene. Further upstream, the resistance genes *floR* and *aph(*3′*)-Ia* were located. However, the IS*6* transposase gene in vicinity to *bla*_TEM-176_ was lacking in our plasmids investigated here. The gene for a hypothetical protein downstream of *qnrS1,* as well as the IS*6* transposase gene downstream of *qnrS1,* were also not detectable. In addition, the IS*6* transposase genes flanking *dfrA* were missing, compared to the reference. The plasmid psg_ww281, as well as the reconstructed plasmids from our in silico analysis, were determined to be self-transmissible, using the mob-suite as the MOB_P_ relaxase and the MPF_T_ mating pair formation type was detected in all psg_ww281-like genomes. The conjugative behaviour of the IncX1 plasmids could be confirmed by laboratory investigations and was determined to be efficient among *E. coli*.

The predominant IncX1 psg_ww281-plasmid is comparable to the aforementioned unnamed IncX1 reference plasmid (NZ_CP020088). The conserved sequence of this plasmid carried the DNA invertase (*hin*) and different components of the type IV secretion system (*ptl*, *vir*). However, in addition to the *qnrS1* and *bla*_TEM_ genes, this plasmid type acquired further resistance determinants, thus, presumably demonstrating the evolution of the plasmids regarding resistance development. Interestingly, the pattern of missing IS*6* elements, compared to the reference, was observed. This can be a result of assembly difficulties in the repeat-rich area of IS elements. It can also present a German counterpart plasmid, compared to the psg_ww281-plasmid. Thus, it would present a plasmid, lacking those IS6 elements and therewith the mobility of the respective resistance genes. As this plasmid type was frequently detected in Europe, it probably represents an important vehicle for resistance progression and should therefore be further monitored.

#### 2.2.5. Characteristics of the Plasmid Assigned to the Unnamed Reference Plasmid of the Strain R1701

**Figure 4:**
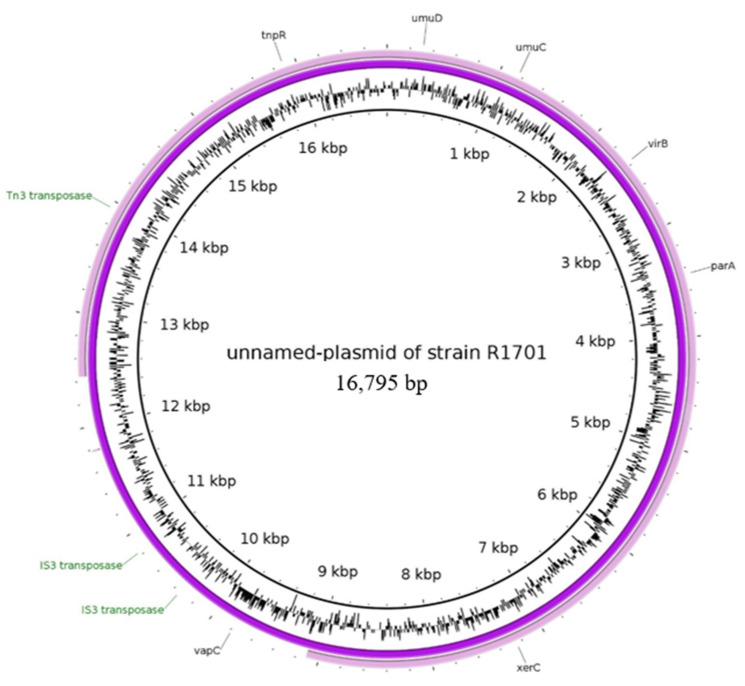
BRIG image of sequence contigs of individual isolates assigned to the unnamed reference plasmid (NZ_CP039972). Contigs belonging to the isolates as follows (from inner to outer ring): unnamed reference plasmid of the strain R1701 and 17-AB01531.

For one isolate, the best-matching reference plasmid was the unnamed plasmid of the strain R1701 (NZ_CP039972). This plasmid exhibited a size of 16,795 bp and did not carry any resistance determinants. The plasmid type was described first in a *Klebsiella pneumoniae* from human blood samples in the USA and seems to be rare, as no further relatives could be detected by blast searches. However, plasmids of larger size ranges with notable similarity to the reference genome were detected by nucleotide comparisons. Furthermore, the unnamed reference plasmid was assigned to the IncR group. When we investigated the contigs of our isolate matching the reference, we found that this plasmid seemed to be evolved into a *qnrS1*- and *bla*_TEM_-carrying plasmid (Figure 4).

**Figure 5:**
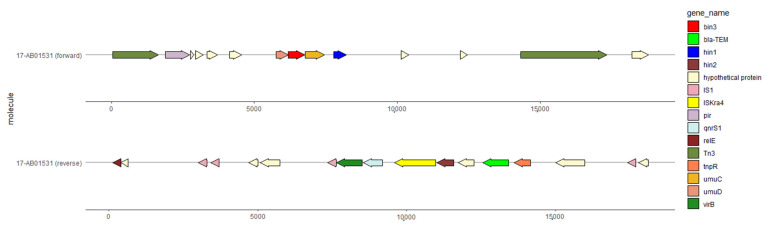
Structural organization of the *qnrS1*-carrying contig of isolate 17-AB01531 derived from mapping against the unnamed reference plasmid (NZ_CP039972). Upper line represents the annotated forward strand. Lower line represents the annotated reverse strand.

The plasmid (NZ_CP039972) carries multiple IS elements. In particular two IS*3* transposase genes were detected. The in-silico generated organization of our plasmid is shown in Figure 5.

The structure presented in Figure 5 carries *qnrS*1 and *bla*_TEM_. It represents the contig not present on the reference plasmid but assigned to it for our plasmid of the strain 17-AB01531. Similarly, to all other plasmid types described in this study, we found the DNA-invertase encoding genes *hin1* and *hin2*. The assignment of the contig to the plasmid led to the co-occurrence of the replicons IncX and IncR. Further in-silico analysis revealed the presence of remnant sequences of a *bla*_TEM_ gene on the reference plasmid. This remnant *bla* sequence was located from 16,346 to 16,795 bp and covers only 52.45% of the *bla*_TEM_ reference gene (NZ_CP039972). Using mob-suite, the reference plasmid was determined to be non-conjugative. In addition, by experimental investigation no conjugative transfer of the plasmid was detected in *E. coli*. 

It is likely that the used reference plasmid does not represent the complete sequence plasmid correctly. As we detected a remnant sequence of the *bla*_TEM_ gene on the genome, it is possible, that the assigned contig sequence (Figure 5) could actually be present on the reference plasmid but not assembled correctly. This suggestion might be supported by the fact, that we could not detect any further plasmid of similar size but found rather larger genomes exhibiting larger DNA regions of high similarity to the reference plasmid. Thus, this type of plasmid might also present a platform for development of resistance gene accumulation. Moreover, the presence of two *inc* groups represents a potential hybrid of two distinct plasmids. This co-occurrence of different *inc* groups has been mentioned before, especially for IncX plasmids. Thus, IncX plasmid sequences were shown to co-integrate within different plasmid genomes, resulting in a broadening of the host range [46]. A project of Slettemeas et al. [1] confirmed this conjugation potential of IncX plasmids and states that these plasmids are successful and widely disseminated. Extending the narrow host range of IncX plasmids to a broader spectrum of potential host bacteria [33,40]. In general, it has been shown that this plasmid type is able to be spread to different species of Enterobacteriaceae. Although *qnrS1*-IncR plasmids had been described before [45], the combined presence of IncX and IncR seems to be rare, as we could not find any description in the current literature.

## 3. Materials and Methods

### 3.1. Isolate Characterization

All *E. coli* recovered during the annual zoonosis monitoring 2017 in Germany, covering the pork and beef production chain, were investigated regarding their minimum inhibitory concentration (MIC) on commercial test plates (EUVSEC/EUVSEC2; Sensititre™, TREK Diagnostic Systems, East Grinstead, UK). MIC values were interpreted according to EUCAST epidemiological cut-off values (ECOFFs) [47]. All isolates determined as being non-wildtype against nalidixic acid (MIC ≥16 µg/mL) and/or ciprofloxacin (MIC ≥ 0.06 µg/mL) were further subjected by PCR for *qnr* gene detection as described according to Cattoir et al. [48]. An extrachromosomal localization of the *qnrS* gene and a size prediction of the plasmid was performed by S1-nuclease pulsed-field gel electrophoresis (S1-PFGE) combined with Southern blotting and DNA-DNA hybridization against a digoxygenin-labelled *qnrS* probe [49]. The phylogenetic relationship of the *qnrS*-carrying *E. coli* was determined by XbaI-macrorestriction PFGE (XbaI-PFGE) in a CHEF-DR III system (Bio-Rad Laboratories, Madrid, Spain) according to the PulseNet standardized laboratory protocol [49]. All *E. coli*, with a confirmed plasmidic localization of *qnrS* were subjected to whole-genome sequencing (WGS).

### 3.2. DNA Extraction and Sequencing

Genomic DNA of *E. coli* was prepared using the PureLink Genomic DNA Mini Kit (Invitrogen-Thermo Fisher, Schwerte, Germany), according to the manufacturer’s recommendation. Sequencing DNA libraries were generated with the Nextera DNA Flex Library Preparation Kit (Illumina^®^, San Diego, CA, USA), as previously described [50]. Short-read, paired end whole-genome sequencing was performed in 2 × 151 cycles using the Illumina^®^ NextSeq™ 500/550 Mid Output Kit v2.5 (300 Cycles). The Unicycler pipeline (version 0.4.4; Wick et al., 2017) recommended for bacterial genomes was used for de novo assembly. Evaluation and quality assessment of genome assemblies were conducted using QUAST 5.0.2 [51]. Assembled contigs were analysed for resistance genes and plasmid markers (i.e., replicon types) with bakcharak [52]. *E. coli* isolates determined to harbor a *qnrS1* gene on a plasmid with the most prevalent replicon type (IncX) were further investigated.

### 3.3. Bioinformatics Analysis, Characterization and Visualization of the WGS Data

To determine the most prevalent *qnrS1* plasmid type, a reference database comprising all available closed *qnr*-plasmid genomes of the Genbank database was developed. Raw reads of all individual isolates were aligned to the genomes of the *qnr*-plasmid database using plasmidID v1.6.5 (https://github.com/BU-ISCIII/plasmidID, accessed on 17 April 2021) to identify the matching reference based on the closest relationship. Further analysis and SNP difference prediction between the estimated reference and the actual investigated plasmid was performed using snippysnake (https://gitlab.com/bfr_bioinformatics/snippySnake, accessed on 17 April 2021).

Visualisation of DNA alignments was done with BRIG [53]. Investigation of similar plasmids was conducted through blast searches [54]. Determination of the multi-locus sequence types (MLST) and the identification of genes involved in antimicrobial resistance development was conducted using the bakcharak pipeline [52]. Annotation of genomes was operated with the annotation tool prokka (v1.14.5) [55]. Phylogenetic relationship of the plasmids was determined with Clustal Omega alignment (v1.2.4) [56] and visualised with iTOL (v6) [57]. Mapping of the corresponding sequences was conducted through visualisation and analysation of the bed-file in geneious (v2020.2.2) [58]. To determine the conjugational transfer of the respective plasmids, we further screened for MOB and MPF components with the mob-suite-tool [59].

### 3.4. Conjugational Test

The transferability of plasmids carrying *qnrS*1 was tested by in vitro filter mating studies. The filter mating experiments were conducted using the plasmid-free, sodium azide-resistant *E. coli* strain J53 as the recipient [60,61]. The conjugative transfer of plasmids was confirmed with S1-PFGE, and PCR as described above. The colonies were stored at −80 °C in a glycerol suspension.

## 4. Conclusions

Here, we determined the predominant *qnrS*1-carrying IncX plasmid types present in commensal and ESBL-producing *E. coli* of the German pork and beef production chain in 2017. Although the *E. coli* harbouring the respective IncX plasmids were highly heterogenic in their characteristics, the prevalent plasmids resemble a predominant genetic basis. In this study, we detected *qnrS1*-carrying IncX1 and IncX3 plasmids that also carried genes for resistance to other antimicrobials, such as *bla*. IncX plasmids seem to represent important carriers for the dissemination of resistance against clinically important antimicrobial agents. A deeper understanding and investigation of the persistence, evolutionary adaption and fitness of the plasmids is highly recommended.

## Figures and Tables

**Table 1:** Characteristics of *E. coli* carrying *qnrS*1 on an IncX plasmid.

Isolate	ST	Resistance Genes *	Source	Phenotypic Resistance Profile
17-AB00542	1288	*aph(*3*′′)-Ib, aph(*6*)-Id, bla*_EC_*, bla*_TEM-1_*, qnrS1, tet*(B)	calf, faeces	TET
17-AB00544	155	*aph(*3*′)-Ia, bla*_EC-18_*, bla*_TEM-176_*, dfrA14, floR*^+^*, qnrS*1*, tet*(A)	calf, faeces	AMP, CHL, CIP, TET, TMP
17-AB00639	10	*aac(*3*)-IVa, aph(*3*″)-Ib, aph(*4*)-Ia, aph(*6*)-Id, bla*_CTX-M-1_*, bla*_EC_*, bla*_TEM-1_*, dfrA5, mph(A), qnrS1, sul2*	pig, faeces	AMP, CIP, FOT, GEN, SMX, TAZ, TMP
17-AB00742	10	*aadA1, bla*_EC-15_*, bla*_TEM-1_*, qnrS*1	pig, faeces	AMP, CIP
17-AB00995	392	*aph(*3*″)-Ib, aph(*6*)-Id, bla*_EC-18_*, bla*_TEM-1_*, qnrS1, sul2, tet*(B)	calf, faeces	AMP, CIP, SMX, TET
17-AB01005	1244	*aadA1, aph(*3*″)-Ib, aph(*6*)-Id, bla*_EC_*, bla*_SHV-12_*, qnrS1, tet*(B)	calf, faeces	FEP, FOT, TAZ
17-AB01006	10	*bla*_EC-15_ *, bla*_SHV-12_ *, qnrS1*	calf, faeces	FEP, FOT, TAZ
17-AB01018	88	*aph(*3*″)-Ib, aph(*3*′)-Ia, aph(*6*)-Id, bla*_EC-13_*, bla*_SHV-12_*, bla*_TEM-1_*, dfrA5, floR, qnrS1, sul2, tet*(A)	pig, faeces	AMP, CHL, CIP, FOT, SMX, TAZ, TET, TMP
17-AB01105	58	*aadA5, bla*_CTX-M-1_*, bla*_EC-18_*, bla*_TEM-1_*, dfrA*17*, dfrA5, qnrS1, sul2, tet*(A)	pig, faeces	FEP, FOT, TAZ
17-AB01352	88	*bla*_EC-13_*, bla*_TEM-1_*, qnrS1, tet*(A)	calf, meat	AMP, CIP, TET
17-AB01531	34	*bla*_EC_ *, bla*_TEM-1_ *, qnrS1, sul2,*	pig, faeces	AMP, CIP, SMX
17-AB01539	10	*aadA1, bla*_EC_ *, bla*_TEM-1_ *, qnrS1*	pig, faeces	AMP, CIP
17-AB01619	10	*aac(*3*)-IIa, aadA5, aph(*3*′)-Ia, bla*_CTX-M-15_*, bla*_EC_*, bla*_TEM-176_*, dfrA14, dfrA17, floR*^+^*, mph*(A), *qacE∆1, qnrS1, sul1, sul2, tet*(A), *tet*(B)	calf, faeces	AMP, AZI, CHL, CIP, FOT, GEN, NAL, SMX, TAZ, TET, TMP
17-AB01686	1288	*aph(*3*″)-Ib, aph(*3*′)-Ia, aph(*6*)-Id, bla*_CTX-M-15_*, bla*_EC_*, bla*_TEM-176_*, dfrA14, floR*^+^*, qnrS1, tet*(A), *tet*(B)	calf, faeces	AMP, CHL, CIP, FOT, SMX, TAZ, TET, TMP
17-AB01707	10	*aph(*3*′)-Ia, bla*_EC_*, bla*_TEM-176_*, dfrA14, floR*^+^*, qnrS1, tet*(A)	calf, faeces	AMP, CHL, CIP, TET, TMP
17-AB01752	641	*aph(*3*″)-Ib, aph(*6*)-Id, bla*_EC-13_*, bla*_TEM-1_*, qnrS1, tet*(B)	pig, faeces	AMP, CIP, TET
17-AB01792	101	*aadA5, bla*_CTX-M-1_ *, bla*_EC-18_ *, bla*_TEM-1_ *, dfrA17, qnrS1, sul2*	pig, faeces	FEP, FOT, TAZ
17-AB01795	10	*bla*_EC-15_ *, bla*_TEM-1_ *, qnrS1*	pig, faeces	AMP, CIP
17-AB01798	641	*aadA1, aadA2, aph(*3*″)-Ib, aph(*6*)-Id, bla*_EC-13_*, bla*_SHV-12_*, bla*_TEM-1_*, cmlA1, dfrA32, ere*(A), *mef*(B), *qacE∆1, qacL, qnrS1, sul1, sul3, tet*(A)	pig, faeces	FEP, FOT, TAZ
17-AB01875	10	*aph(*3*′)-Ia, bla*_CTX-M-1_*, bla*_EC_*, bla*_TEM-176_*, dfrA14, floR*^+^*, mph*(A), *qnrS1, tet*(A)	calf, faeces	AMP, CHL, CIP, FOT, TAZ, TET, TMP
17-AB01969	711	*bla*_EC-18_*, bla*_TEM-1_*, mph*(A), *mph*(E), *msr*(E), *qnrS1*	calf, faeces	AMP, AZI, CIP
17-AB02071	58	*bla*_EC-18_ *, bla*_SHV-12_ *, qnrS1*	calf, faeces	AMP, CIP, FOT, TAZ
17-AB02090	48	*bla*_CTX-M-1_ *, bla*_EC-15_ *, bla*_TEM-1_ *, qnrS1*	calf, faeces	AMP, CIP, FOT, TAZ
17-AB02355	2230	*aadA1, aadA2, aph(*6*)-Id, bla*_EC-13_*, bla*_SHV-12_*, bla*_TEM-1_*, cmlA1, dfrA14, qacL, qnrS1, sul2, sul3, tet*(A)	pig, faeces	AMP, CIP, FOT, SMX, TAZ, TET, TMP
17-AB02707	58	*aac(*3*)-IVa, aph(*3*″)-Ib, aph(*6*)-Id, bla*_CTX-M-1_*, bla*_EC-18_*, bla*_TEM-1_*, qnrS1*	calf, faeces	AMP, CIP, FOT, TAZ
17-AB02711	7469	*aadA2, aph(*3*′)-Ia, bla*_CTX-M-1_*, bla*_EC-8_*, bla*_TEM-176_*, dfrA14, floR*^+^*, lnu*(F), *mph*(A), *qnrS1, tet*(A)	calf, faeces	AMP, CIP, FOT, SMX, TAZ, TET, TMP
17-AB02721	7469	*aadA2, aph(*3*′)-Ia, bla*_CTX-M-1_*, bla*_EC-8_*, bla*_TEM-176_*, dfrA14, floR*^+^*, lnu*(F), *mph*(A), *qnrS1, tet*(A)	calf, faeces	FEP, FOT, TAZ
17-AB02726	7469	*aadA2, aph(*3*′)-Ia, bla*_CTX-M-1_*, bla*_EC-8_*, bla*_TEM-147_*, dfrA14, floR*^+^*, lnu*(F), *mph*(A), *qnrS1, tet*(A)	calf, faeces	FEP, FOT, TAZ
17-AB02951	2496	*aadA1, aadA5, aph(*3*″)-Ib, aph(*6*)-Id, bla*_CTX-M-1_*, bla*_EC-15_*, bla*_TEM-1_*, dfrA1, dfrA17, mef*(C), *mph*(B)*, mph*(G), *qac∆1, qnrS1, sul1, sul2*	pig, faeces	FEP, FOT, TAZ

^+^ Identity below 100%; * If possible, the variant for *bla*_EC_-like genes is given. However, for some genes the variant could not be determined. Abbreviations: AMP = ampicillin, CHL = chloramphenicol, CIP = ciprofloxacin, FOT = cefotaxime, GEN = gentamicin, NAL = nalidixic acid, SMX = sulfamethoxazole, TAZ = ceftazidime, TET = tetracycline, TMP = trimethoprim.

**Table 2:** Characteristics of best-matching IncX plasmids carrying *qnrS*1 with aligned neighbour-joining tree without distance corrections of all four best matching reference plasmids based on Clustal Omega multiple sequence alignment.

	Plasmid Name	Reference Plasmid	AMR Genes	Inc	bp
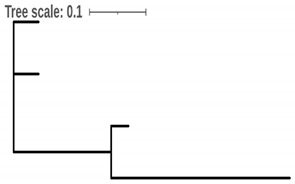	unnamed plasmid of strain R17071	NZ_CP039972.1	*bla*_TEM_ *	IncR, IncX1	16,795
	pKpvST101_6	NZ_CP031373.1	*bla*_SHV_ *, qnrS1*	IncX3	43,670
	unnamed plasmid of strain 0670	NZ_CP020088.1	*bla*_TEM-1_ *, qnrS1*	IncX1, IncX3	47,674
	psg_ww281 plasmid	NZ_CP037995.1	*aph(*3*′)-Ia, bla*_TEM-176_*, dfrA14, floR, qnrS1, tet*(A)	IncX1	48,223

* a *bla*_TEM_ derivative was detected but is only covered by 52.45%.
